# What *Health Equity* Journal Stands For

**DOI:** 10.1089/heq.2025.0031

**Published:** 2025-03-11

**Authors:** Monica R. McLemore, on behalf of the Associate Editors of Health Equity Journal

**Affiliations:** Child, Family and Population Health Nursing, University of Washington, Seattle, Washington, USA.

At the beginning of 2025, chaos and panic marked the start of the second Trump administration because of executive orders^1–2^ that stifled much of the scientific infrastructure in the United States. The lack of preparedness for the dismantling of equity, diversity, and inclusion initiatives^3^ was unacceptable—given the slate of nominees for cabinet-level positions, the purpose and function of the Department of Government Efficiency^4^ and Project 2025^5^—which all provided a preview of the direction the administration would take specific to health and human services. Unfortunately, many impacted entities, namely universities, grantees, federal agencies, hospitals, health systems, and professional organizations, rapidly complied with these executive orders, despite the question of their legality. Anticipatory compliance undermines our ability to maintain progress and engage in resistance through the protections afforded by our constitutional rights.^6^ In light of these realities, we at *Health Equity (HEQ)* journal want to be very clear about who we are, what we stand for, and how we expect to move in this rapidly changing environment.

First, as Editor in Chief, it is my responsibility to ensure that evidence-based research that we publish is both accurate and ethical. Our editorial board is committed to these principles and will continue to publish high-quality manuscripts that reflect the most current scientific ideas and language, regardless of if they appear on banned lists or other edicts that are incongruent with academic freedom.

Second, *HEQ* is a peer-reviewed open access journal that addresses the urgent need for authoritative information about health disparities and health equity among vulnerable populations. We accept multiple definitions of health equity in manuscripts we publish, and the most appropriate for this current moment that is guiding the work of the journal is the one from Dr. Camara Jones,^7^ which defines health equity as “the assurance of the condition of optimal health for all people.” Given this definition, we see no reason to change anything about the current operations of the journal.

Third, *HEQ* ultimately plans to publish the research as the investigators intended. Whether or not authors choose to comply with whatever institutional restrictions on language, concepts, and measures, we will insist on accurate science and appropriate citational practices. To this end, we offer clear guidance below for both new and seasoned authors to expedite the peer review process in these dynamic times:

- Authors will be responsible for checking that any reference to federal websites, URLs, and other documents continue to be accurate. We recommend the use of the Internet Archive (also known as the Wayback Machine, see https://web.archive.org/).
- The *HEQ* editorial board will begin to use a new tool developed using funding from the American Nurses Association and the National Commission to Address Racism in Nursing.
- Any past cited science will not be modified to comply with current executive orders or mandates. In other words, we will honor and use the language in the historical published record and request scientific accuracy from all authors. No doubt, the peer review process will be impacted by the higher level of scrutiny to maintain scientific accuracy and integrity. We appreciate your patience as we navigate the new landscape.

As a reminder, we will continue to accept reports of original research, review articles, narrative reviews, book reviews, short reports, perspectives, editorials, and letters to the editor. It is important in this moment to remind our community that we continue to value the work of early career-investigators and see the development of new authors as a key value of *HEQ*.

Finally, I want to share exciting news about our Supporting Experts By Experience: Community Voice in scientific publications (SEE Community Voice project). In the next 2 years, we will be rolling out programming that will allow for deeper community partnership in scientific publishing, including special topic issues, roundtables, and the piloting of a national citizen science/community peer review board to participate in our peer review. Hopefully greater involvement of the people who fund public science and those who benefit from our discoveries will restore trust in science and combat mis- and disinformation.

My disciplinary orientation as a nurse provides me clear guidance in this moment. Our social contract and code of ethics support a life of service to the public and adherence to professional standards. As always, I’m grateful to serve in the role of Editor in Chief and welcome your comments, feedback, and suggestions. You can find me @mclemoremr on most social media platforms and can email me through the ScholarOne portal (https://home.liebertpub.com/publications/health-equity/641/editorial-board).

## Note Added After Publication

A supplemental file that was originally published with this Editorial has been removed as it was not intended for publication.

## Abbreviations Used

HEQ: Health Equity Journal
SEE: Supporting Experts by Experience
URL: Uniform Resource Locator
