# *Correction to:* What *Health Equity* Stands For (doi: 10.1089/heq.2025.0031)

**DOI:** 10.1089/heq.2025.0031.correx

**Published:** 2025-03-31

A supplemental file named, “Guidelines for Conducting an Equity Review: Statement of Purpose” has been removed from the editorial entitled, “What *Health Equity* Stands For” by Monica R. McLemore on behalf of the Associate Editors of the *Health Equity* Journal (Health Equity, 9(1):142–143; doi: 10.1089/heq.2025.0031) as it was published erroneously. It was intended only for the peer review process and not intended for publication.

The Publisher of *Health Equity* regrets this error and apologizes to the authors and readers of the journal.

